# Crystal structure of 1,1′-{(pentane-1,5-di­yl)bis[(aza­niumylyl­idene)methanylyl­idene]}bis(naphthalen-2-olate)

**DOI:** 10.1107/S2056989015014437

**Published:** 2015-08-06

**Authors:** Kamel Ouari, Moufida Merzougui, Lydia Karmazin

**Affiliations:** a Laboratoire d’lectrochimie, d’Ingénierie Moléculaire et de Catalyse Redox, Faculty of Technology, University of Ferhat Abbas Sétif-1, 19000 Sétif, Algeria; b Service de Radiocristallographie, Institut de Chimie UMR 7177 CNRS-Université de Strasbourg, 1 rue Blaise Pascal, BP296/R8, 67008 Strasbourg Cedex, France

**Keywords:** crystal structure, 1,5-di­amino­pentane, 2-hy­droxy-1-naphthaldehyde, zwitterion, bis-zwitterion, hydrogen bonding

## Abstract

The whole mol­ecule of the title compound, C_27_H_26_N_2_O_2_, is generated by twofold rotational symmetry, with the central C atom of the pentyl chain located on the twofold rotation axis. The compound crystallizes as a bis-zwitterion, and there are two intra­molecular N—H⋯O hydrogen bonds generating *S*(6) ring motifs. In the crystal, mol­ecules are linked by pairs of C—H⋯O hydrogen bonds, forming ribbons propagating along [001], and enclosing *R*_2_^2^(22) ring motifs.

## Abstract 2

The whole mol­ecule of the title Schiff base is generated by twofold rotational symmetry. It crystallizes as a bis-zwitterion, and there are two intra­molecular N—H⋯O hydrogen bonds present. In the crystal, mol­ecules are linked by pairs of C—H⋯O hydrogen bonds forming ribbons propagating along [001].

## Chemical context   

Tetra­dentate NNOO Schiff-bases have been used extensively as supporting ligands in *d*-block chemistry because of their ability to stabilize metals in various oxidation states (Alaghaz *et al.*, 2014 ▸; Kianfar *et al.*, 2015 ▸; Mikhalyova *et al.*, 2014 ▸; Borthakur *et al.*, 2014 ▸; Basumatary *et al.*, 2015 ▸). For many years, particular attention has been devoted to imines because of their uses as catalysts in various organic transformations (Khorshidifard *et al.*, 2015 ▸), and for their anti­cancer (Shiju *et al.*, 2015 ▸), anti­fungal (Abo-Aly *et al.*, 2015 ▸) and anti­bacterial (Salehi *et al.*, 2015 ▸) properties. They have also been used as sensors (Bandi *et al.*, 2013 ▸), corrosion inhibitors (Dasami *et al.*, 2015 ▸) and optical and fluorescent probes (Shoora *et al.*, 2015 ▸; Prabhakara *et al.*, 2015 ▸). 
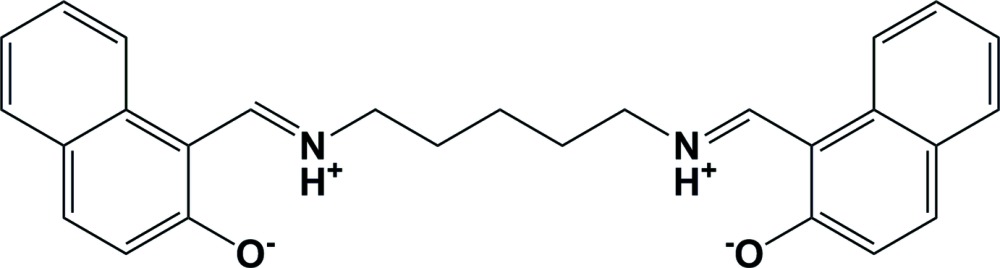


The microwave-assisted synthesis method, in solvent or solvent-free, is efficient and rapid. It gives cleaner reactions, is ease to use, gives higher yields and is a more economical synthetic process for the preparation of Schiff base compounds compared to conventional methods. It has been used to enhance the yield and reduce the time of certain reactions: for example, a one-step synthesis of D-A-D chromo­phores as active materials for organic solar cells (Jeux *et al.*, 2015 ▸), or the synthesis of a series of acyclic Schiff base–chromium(III) complexes (Kumar *et al.*, 2015 ▸).

In a continuation of our work on Schiff base ligands, we report herein on the crystal structure of the title compound, synthesized using two methods, *viz*. microwave irradiation and conventional, by condensing *o*-hy­droxy­naphthaldehyde and 1,5-di­amino­pentane.

## Structural commentary   

**Figure 1:**
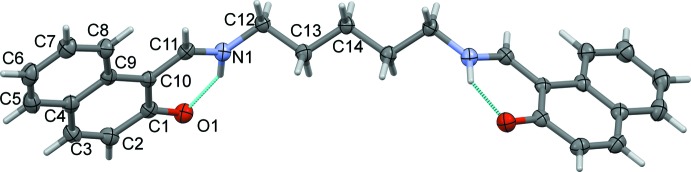
The mol­ecular structure of the title compound, with atom labelling. Displacement ellipsoids are drawn at the 50% probability level. The intra­molecular hydrogen bonds are shown as dashed lines (see Table 1 ▸). The unlabelled atoms are related to the labelled atoms by twofold rotational symmetry (atom C14 lies on the twofold axis; symmetry code: −*x*, *y*, −*z* + 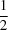).

The whole mol­ecule of the title compound, Fig. 1 ▸, is generated by twofold rotational symmetry, with the central C atom of the pentyl chain, C14, located on the twofold rotation axis. It crystallizes as a bis-zwitterion, with strong intra­molecular N—H⋯O hydrogen bonding between the imino N atom N1 (N1^’^), and the O atom, O1 (O1^i^) [*d* (O⋯N) = 2.5437 (17) Å; symmetry code: (i) −*x*, y, −*z* + 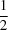], forming *S*(6) ring motifs (Fig. 1 ▸ and Table 1 ▸). The pentyl chain has an extended conformation with the naphthalene rings inclined to one another by 89.94 (5)°.

## Supra­molecular features   

**Figure 2:**
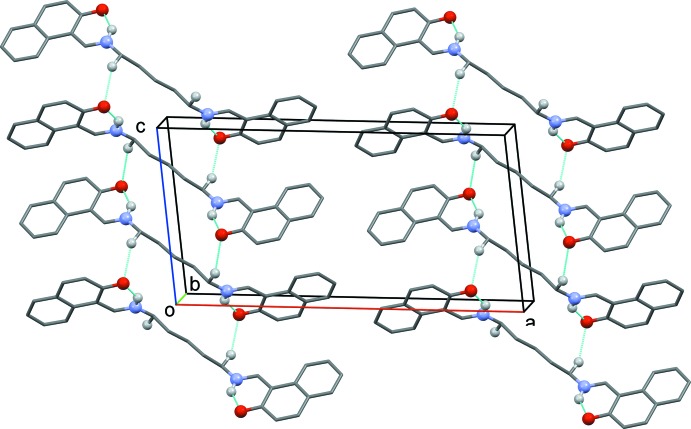
Crystal packing of the title compound viewed along the *b* axis. The hydrogen bonds are shown as dashed lines (see Table 1 ▸). For clarity, only the H atoms involved in hydrogen bonding have been included.

In the crystal, mol­ecules are linked by pairs of C—H⋯O hydrogen bonds, forming ribbons propagating along [001] and enclosing 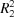
(22) ring motifs (Table 1 ▸ and Fig. 2 ▸).

## Database survey   

Recently, our group reported the crystal structures of three new Schiff bases synthesized using conventional or ultrasonic irradiation methods by reacting primary amines and *o*-hy­droxy­naphthaldehyde (Ouari *et al.*, 2015*a* ▸,*b* ▸,*c* ▸). They too crystallize as bis-zwitterionic compounds with strong intra­molecular N—H⋯O hydrogen bonds forming *S*(6) ring motifs.

## Synthesis and crystallization   

**Method 1: Microwave synthesis**

2-Hy­droxy-1-naphthaldehyde (0.344g, 2 mmol), mixed and ground in a mortar, was placed in a reaction flask, and then 1,5-di­amino­pentane (0.109 g, 1 mmol) in 2 ml of methanol was added. The reaction mixture was then irradiated in a microwave oven for 1 min at 600 W. Upon completion, based on TLC analysis (silica gel, CH_2_Cl_2_/MeOH, 9.5/0.5, *v*/*v*), the product was washed with methanol (3 × 3 ml) and diethyl ether (3 × 3 ml) and filtered. Yellow crystals of the title compound, suitable for X-ray diffraction analysis, were obtained after two days by slow evaporation of a solution in DMSO/MeOH (yield: 95%, m.p.: 438–440 K). Elemental analysis calculated for C_27_H_26_N_2_O_2_: C, 80.00; H, 6.38; N,6.82%; found: C, 80.42; H, 6.63; N, 6.56%.

**Method 2: Conventional synthesis**

The title Schiff base was prepared by condensation between 1,5-di­amino­pentane (51 mg, 0.5 mmol) and 2-hy­droxy-1-naphthaldehyde (172 mg, 1 mmol) in methanol (10 ml). The mixture was refluxed and stirred under a nitro­gen atmosphere for 3 h. The precipitate obtained was filtered, washed with methanol and diethyl ether and dried in vacuum overnight. Yellow single crystals of the title compound were obtained by slow evaporation of a solution in methanol (yield 71%; m.p.: 438–440 K).

As expected, the yield using method 1 (95%) is significantly greater than that using method 2 (71%).

## Refinement   

Crystal data, data collection and structure refinement details are summarized in Table 2 ▸. The iminium H atom was located from a difference Fourier map and freely refined. C-bound H atoms were included in calculated positions and treated as riding atoms: C—H = 0.95 − 0.99 Å with *U*_iso_(H) = 1.2*U*_eq_(C). Atom C14 lies on the twofold rotation axis and the H atoms were placed using instruction HFIX 23 (Sheldrick, 2015 ▸); the occupancy of the methyl­ene H atoms were fixed automatically at 0.5.

## Supplementary Material

Crystal structure: contains datablock(s) I, Global. DOI: 10.1107/S2056989015014437/su5181sup1.cif

Structure factors: contains datablock(s) I. DOI: 10.1107/S2056989015014437/su5181Isup2.hkl

CCDC reference: 1416064

Additional supporting information:  crystallographic information; 3D view; checkCIF report

## Figures and Tables

**Table 1:** Hydrogen-bond geometry (, )

*D*H*A*	*D*H	H*A*	*D* *A*	*D*H*A*
N1H1*N*O1	0.96(2)	1.72(2)	2.5437(17)	141.3(16)
C12H12*A*O1^i^	0.99	2.45	3.2871(19)	142

Symmetry code: (i) 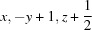.

**Table 2:** Experimental details

Crystal data	
Chemical formula	C_27_H_26_N_2_O_2_
*M*_r_	410.50
Crystal system, space group	Monoclinic, *P*2/*c*
Temperature (K)	173
*a*, *b*, *c* ()	20.9080(13), 4.7429(2), 10.6810(6)
()	96.419(3)
*V* (^3^)	1052.54(10)
*Z*	2
Radiation type	Mo *K*
(mm^1^)	0.08
Crystal size (mm)	0.45 0.20 0.10

Data collection	
Diffractometer	Nonius KappaCCD
Absorption correction	Multi-scan (*MULSCAN* in *PLATON*; Spek, 2009 ▸)
*T*_min_, *T*_max_	0.792, 1.000
No. of measured, independent and observed [*I* > 2(*I*)] reflections	5781, 1958, 1402
*R*_int_	0.049
(sin /)_max_ (^1^)	0.606

Refinement	
*R*[*F* ^2^ > 2(*F* ^2^)], *wR*(*F* ^2^), *S*	0.049, 0.120, 1.08
No. of reflections	1958
No. of parameters	146
H-atom treatment	H atoms treated by a mixture of independent and constrained refinement
_max_, _min_ (e ^3^)	0.16, 0.14

Computer programs: *COLLECT* (Nonius, 1998 ▸), *DENZO* and *SCALEPACK* (Otwinowski Minor, 1997 ▸), *SHELXS2014* (Sheldrick, 2008 ▸), *SHELXL2014* (Sheldrick, 2015 ▸), *Mercury* (Macrae *et al.*, 2008 ▸) and *PLATON* (Spek, 2009 ▸).

## supplementary crystallographic information

### Crystal data

**Table:**

C_27_H_26_N_2_O_2_	*F*(000) = 436
*M**_r_* = 410.50	*D*_x_ = 1.295 Mg m^−^^3^
Monoclinic, *P*2/*c*	Mo *K*α radiation, λ = 0.71073 Å
*a* = 20.9080 (13) Å	Cell parameters from 7575 reflections
*b* = 4.7429 (2) Å	θ = 1.0–27.5°
*c* = 10.6810 (6) Å	µ = 0.08 mm^−^^1^
β = 96.419 (3)°	*T* = 173 K
*V* = 1052.54 (10) Å^3^	Plate, yellow
*Z* = 2	0.45 × 0.20 × 0.10 mm

### Data collection

**Table:**

Nonius KappaCCD diffractometer	1402 reflections with *I* > 2σ(*I*)
Radiation source: fine-focus sealed tube	*R*_int_ = 0.049
phi and ω scans	θ_max_ = 25.5°, θ_min_ = 2.9°
Absorption correction: multi-scan (MULSCAN in *PLATON*; Spek, 2009)	*h* = −25→23
*T*_min_ = 0.792, *T*_max_ = 1.000	*k* = −5→5
5781 measured reflections	*l* = −12→8
1958 independent reflections	

### Refinement

**Table:**

Refinement on *F*^2^	Secondary atom site location: difference Fourier map
Least-squares matrix: full	Hydrogen site location: mixed
*R*[*F*^2^ > 2σ(*F*^2^)] = 0.049	H atoms treated by a mixture of independent and constrained refinement
*wR*(*F*^2^) = 0.120	*w* = 1/[σ^2^(*F*_o_^2^) + (0.0549*P*)^2^ + 0.0593*P*] where *P* = (*F*_o_^2^ + 2*F*_c_^2^)/3
*S* = 1.08	(Δ/σ)_max_ < 0.001
1958 reflections	Δρ_max_ = 0.16 e Å^−^^3^
146 parameters	Δρ_min_ = −0.14 e Å^−^^3^
0 restraints	Extinction correction: *SHELXL2014* (Sheldrick, 2015), Fc^*^=kFc[1+0.001xFc^2^λ^3^/sin(2θ)]^-1/4^
Primary atom site location: structure-invariant direct methods	Extinction coefficient: 0.033 (7)

### Special details

**Table:**

Geometry. All e.s.d.'s (except the e.s.d. in the dihedral angle between two l.s. planes) are estimated using the full covariance matrix. The cell e.s.d.'s are taken into account individually in the estimation of e.s.d.'s in distances, angles and torsion angles; correlations between e.s.d.'s in cell parameters are only used when they are defined by crystal symmetry. An approximate (isotropic) treatment of cell e.s.d.'s is used for estimating e.s.d.'s involving l.s. planes.

### Fractional atomic coordinates and isotropic or equivalent isotropic displacement parameters (Å^2^)

**Table:**

	*x*	*y*	*z*	*U*_iso_*/*U*_eq_	Occ. (<1)
O1	0.14942 (5)	0.8929 (2)	−0.10611 (10)	0.0508 (4)	
N1	0.13436 (6)	0.5012 (3)	0.05060 (12)	0.0436 (4)	
H1N	0.1201 (8)	0.643 (4)	−0.0110 (19)	0.080 (6)*	
C1	0.21054 (7)	0.8695 (3)	−0.07692 (14)	0.0412 (4)	
C2	0.25371 (8)	1.0411 (3)	−0.13960 (15)	0.0497 (5)	
H2	0.2364	1.1708	−0.2022	0.060*	
C3	0.31790 (9)	1.0235 (3)	−0.11224 (17)	0.0546 (5)	
H3	0.3446	1.1413	−0.1562	0.065*	
C4	0.34731 (8)	0.8329 (3)	−0.01900 (15)	0.0469 (4)	
C5	0.41450 (8)	0.8204 (4)	0.00866 (18)	0.0612 (5)	
H5	0.4407	0.9383	−0.0362	0.073*	
C6	0.44291 (8)	0.6423 (4)	0.09873 (19)	0.0632 (5)	
H6	0.4884	0.6365	0.1169	0.076*	
C7	0.40413 (8)	0.4698 (4)	0.16322 (18)	0.0589 (5)	
H7	0.4235	0.3446	0.2258	0.071*	
C8	0.33846 (7)	0.4769 (3)	0.13821 (16)	0.0508 (5)	
H8	0.3132	0.3566	0.1841	0.061*	
C9	0.30745 (7)	0.6580 (3)	0.04622 (14)	0.0403 (4)	
C10	0.23834 (7)	0.6743 (3)	0.01610 (13)	0.0377 (4)	
C11	0.19669 (7)	0.4956 (3)	0.07450 (14)	0.0405 (4)	
H11	0.2152	0.3635	0.1348	0.049*	
C12	0.09067 (7)	0.3217 (3)	0.11165 (15)	0.0440 (4)	
H12A	0.1159	0.1903	0.1699	0.053*	
H12B	0.0644	0.2083	0.0472	0.053*	
C13	0.04669 (7)	0.4962 (3)	0.18441 (15)	0.0454 (4)	
H13A	0.0219	0.6281	0.1257	0.054*	
H13B	0.0733	0.6100	0.2482	0.054*	
C14	0.0000	0.3185 (4)	0.2500	0.0449 (6)	
H14A	−0.0247	0.1956	0.1871	0.054*	0.5
H14B	0.0247	0.1955	0.3129	0.054*	0.5

### Atomic displacement parameters (Å^2^)

**Table:**

	*U*^11^	*U*^22^	*U*^33^	*U*^12^	*U*^13^	*U*^23^
O1	0.0484 (7)	0.0583 (7)	0.0443 (7)	0.0043 (5)	−0.0014 (5)	0.0039 (5)
N1	0.0412 (8)	0.0500 (8)	0.0395 (8)	0.0013 (6)	0.0038 (6)	0.0011 (6)
C1	0.0460 (9)	0.0454 (9)	0.0314 (8)	0.0001 (7)	0.0015 (7)	−0.0083 (7)
C2	0.0615 (12)	0.0473 (9)	0.0399 (10)	−0.0025 (8)	0.0040 (8)	0.0025 (7)
C3	0.0581 (11)	0.0563 (10)	0.0504 (11)	−0.0114 (8)	0.0112 (9)	0.0009 (8)
C4	0.0462 (10)	0.0501 (10)	0.0442 (10)	−0.0049 (8)	0.0049 (7)	−0.0107 (8)
C5	0.0470 (11)	0.0745 (12)	0.0629 (12)	−0.0127 (9)	0.0096 (9)	−0.0054 (10)
C6	0.0395 (10)	0.0817 (13)	0.0674 (13)	−0.0006 (9)	0.0010 (9)	−0.0129 (11)
C7	0.0472 (10)	0.0683 (12)	0.0587 (12)	0.0052 (9)	−0.0051 (9)	−0.0027 (9)
C8	0.0434 (10)	0.0578 (10)	0.0501 (11)	0.0005 (8)	0.0008 (8)	−0.0002 (8)
C9	0.0416 (9)	0.0435 (9)	0.0356 (9)	−0.0006 (7)	0.0042 (7)	−0.0103 (7)
C10	0.0402 (8)	0.0405 (8)	0.0322 (8)	−0.0008 (7)	0.0038 (7)	−0.0059 (6)
C11	0.0407 (9)	0.0446 (9)	0.0352 (9)	0.0049 (7)	−0.0002 (7)	−0.0047 (7)
C12	0.0405 (9)	0.0463 (9)	0.0450 (10)	−0.0027 (7)	0.0035 (7)	−0.0002 (7)
C13	0.0415 (9)	0.0479 (9)	0.0467 (10)	−0.0006 (7)	0.0049 (7)	0.0007 (7)
C14	0.0375 (12)	0.0466 (12)	0.0502 (14)	0.000	0.0023 (10)	0.000

### Geometric parameters (Å, º)

**Table:**

O1—C1	1.2858 (17)	C7—C8	1.369 (2)
N1—C11	1.2999 (19)	C7—H7	0.9500
N1—C12	1.4551 (19)	C8—C9	1.408 (2)
N1—H1N	0.96 (2)	C8—H8	0.9500
C1—C10	1.433 (2)	C9—C10	1.447 (2)
C1—C2	1.435 (2)	C10—C11	1.410 (2)
C2—C3	1.344 (2)	C11—H11	0.9500
C2—H2	0.9500	C12—C13	1.515 (2)
C3—C4	1.432 (2)	C12—H12A	0.9900
C3—H3	0.9500	C12—H12B	0.9900
C4—C5	1.404 (2)	C13—C14	1.5191 (18)
C4—C9	1.413 (2)	C13—H13A	0.9900
C5—C6	1.365 (3)	C13—H13B	0.9900
C5—H5	0.9500	C14—C13^i^	1.5190 (18)
C6—C7	1.388 (3)	C14—H14A	0.9900
C6—H6	0.9500	C14—H14B	0.9900
			
C11—N1—C12	124.46 (14)	C8—C9—C4	116.82 (14)
C11—N1—H1N	112.0 (11)	C8—C9—C10	123.95 (14)
C12—N1—H1N	123.5 (11)	C4—C9—C10	119.23 (14)
O1—C1—C10	122.62 (14)	C11—C10—C1	118.19 (14)
O1—C1—C2	119.85 (14)	C11—C10—C9	121.36 (14)
C10—C1—C2	117.52 (14)	C1—C10—C9	120.43 (13)
C3—C2—C1	121.89 (16)	N1—C11—C10	123.79 (14)
C3—C2—H2	119.1	N1—C11—H11	118.1
C1—C2—H2	119.1	C10—C11—H11	118.1
C2—C3—C4	122.09 (16)	N1—C12—C13	110.97 (12)
C2—C3—H3	119.0	N1—C12—H12A	109.4
C4—C3—H3	119.0	C13—C12—H12A	109.4
C5—C4—C9	120.18 (16)	N1—C12—H12B	109.4
C5—C4—C3	120.99 (16)	C13—C12—H12B	109.4
C9—C4—C3	118.83 (15)	H12A—C12—H12B	108.0
C6—C5—C4	121.37 (17)	C12—C13—C14	113.06 (12)
C6—C5—H5	119.3	C12—C13—H13A	109.0
C4—C5—H5	119.3	C14—C13—H13A	109.0
C5—C6—C7	118.82 (17)	C12—C13—H13B	109.0
C5—C6—H6	120.6	C14—C13—H13B	109.0
C7—C6—H6	120.6	H13A—C13—H13B	107.8
C8—C7—C6	121.16 (18)	C13^i^—C14—C13	112.58 (17)
C8—C7—H7	119.4	C13^i^—C14—H14A	109.1
C6—C7—H7	119.4	C13—C14—H14A	109.1
C7—C8—C9	121.65 (16)	C13^i^—C14—H14B	109.1
C7—C8—H8	119.2	C13—C14—H14B	109.1
C9—C8—H8	119.2	H14A—C14—H14B	107.8
			
O1—C1—C2—C3	−179.92 (15)	C3—C4—C9—C10	0.6 (2)
C10—C1—C2—C3	−0.6 (2)	O1—C1—C10—C11	2.0 (2)
C1—C2—C3—C4	0.0 (3)	C2—C1—C10—C11	−177.25 (12)
C2—C3—C4—C5	−179.42 (16)	O1—C1—C10—C9	−179.47 (13)
C2—C3—C4—C9	0.0 (2)	C2—C1—C10—C9	1.2 (2)
C9—C4—C5—C6	−0.4 (3)	C8—C9—C10—C11	−3.1 (2)
C3—C4—C5—C6	179.02 (17)	C4—C9—C10—C11	177.16 (13)
C4—C5—C6—C7	0.4 (3)	C8—C9—C10—C1	178.51 (14)
C5—C6—C7—C8	−0.3 (3)	C4—C9—C10—C1	−1.3 (2)
C6—C7—C8—C9	0.2 (3)	C12—N1—C11—C10	−178.86 (13)
C7—C8—C9—C4	−0.2 (2)	C1—C10—C11—N1	−1.3 (2)
C7—C8—C9—C10	−179.94 (15)	C9—C10—C11—N1	−179.82 (13)
C5—C4—C9—C8	0.3 (2)	C11—N1—C12—C13	117.93 (15)
C3—C4—C9—C8	−179.15 (13)	N1—C12—C13—C14	179.96 (11)
C5—C4—C9—C10	−179.95 (14)	C12—C13—C14—C13^i^	−176.30 (15)

Symmetry code: (i) −*x*, *y*, −*z*+1/2.

### Hydrogen-bond geometry (Å, º)

**Table:**

*D*—H···*A*	*D*—H	H···*A*	*D*···*A*	*D*—H···*A*
N1—H1*N*···O1	0.96 (2)	1.72 (2)	2.5437 (17)	141.3 (16)
C12—H12*A*···O1^ii^	0.99	2.45	3.2871 (19)	142

Symmetry code: (ii) *x*, −*y*+1, *z*+1/2.
